# Valgus sliding subtrochanteric osteotomy for neglected fractures of the proximal femur; surgical technique and a retrospective case series

**DOI:** 10.1186/1749-799X-8-4

**Published:** 2013-03-15

**Authors:** Ashok S Gavaskar, Naveen T Chowdary

**Affiliations:** 1 Dept of trauma, Parvathy hospital, Chennai, India; 2 Dhruv clinics, Chennai, India

## Abstract

**Background:**

Conventional technique of valgus osteotomy of the proximal femur involves removal of a partial or full thickness lateral based wedge from the peritrochanteric region. The purpose of this article is to describe a novel technique of valgus subtrochanteric osteotomy for proximal femur nonunion.

**Methods:**

11 patients with proximal femur nonunions {intracapsular fractures – 7, extracapsular fractures – 4} were treated using a new technique of sliding subtrochanteric osteotomy and DHS fixation. Outcomes analysed include radiological outcome in terms of improvement in Pauwel’s angle, neck-shaft angle and evidence of radiological union at the nonunion site and osteotomy site. Other outcomes analysed include, measurement of limb length discrepancy and functional outcome assessment with Oxford hip score.

**Results:**

Union at the nonunion site and the osteotomy site was achieved in all patients. There were significant improvements in the postoperative Pauwel’s angle, neck shaft angle and Oxford hip score. Limb length discrepancy improved to less than 1 cm in all patients. There was no x ray evidence of avascular necrosis of the femoral head at one year follow-up.

**Conclusions:**

The sliding osteotomy technique is simple, does not need extensive pre operative planning or removal of bone from the proximal femur.

## Introduction

Proximal femur nonunion in a young patient is difficult to treat. While the option of a prosthetic joint may be a straight forward solution in an older patient, preservation of the femoral head is ideal in young individuals. While nonunion is common in intracapsular fractures, extracapsular fractures rarely fail to unite. Osteosynthesis in a femoral neck nonunion is challenging because of the fracture morphology and augmentation techniques in the form of grafting or osteotomies are often required. A proximal femur nonunion represents a biomechanical failure rather than a biological failure alone. So restoration of biomechanics at the proximal femur and at the nonunion site is considered an integral part of any surgical procedure aimed at achieving successful osteosynthesis.

A valgus angulation osteotomy improves the fracture biomechanics by making the fracture plane more horizontal thereby enabling compression at the nonunion site. It also helps in improving limb length and restores the neck shaft angle. Several authors have reported success with the procedure [1,2]. Techniques described previously involve removal of either a full or partial thickness laterally based wedge at the intertrochanteric or subtrochanteric region.

We report on a novel technique of valgus subtrochanteric osteotomy without wedge removal, which was successfully carried out in 11 young patients with an ununited fracture of the proximal femur at a tertiary care center.

## Patients and methods

We operated upon 11 patients with ununited fractures of the neck of femur {intracapsular fractures – 7, extracapsular fractures – 4} at our institution using valgus sliding subtrochanteric osteotomy and DHS fixation. The study was approved by the institutional review board (ref: POH/LL/T/063) and informed consent was obtained from all patients. The average age was 41 {31–55 years} and all were males. The average duration between the index injury and surgery was 6 months {5 – 9 months}. 9 patients underwent native treatment with serial casts and 2 patients underwent no treatment. All patients complained of pain and were restricted to movement with crutches or canes within their domestic environment. Clinical examination revealed a mean shortening of 1.6 cm {1 – 2.5 cm}. All patients filled up the Oxford hip score questionnaire {adapted for the local language} to ascertain their level of function before surgery. Traction and internal rotation radiographs were taken as part of the initial evaluation *to ascertain the neck shaft angle with traction and the Pauwel’s angle*. 4 fractures were categorized as Pauwel’s type III and 7 fractures as Pauwel’s type II.

### Surgical technique

*No extensive pre operative sketching or templating was done. It was decided to use a 135 degree barrel plate in all patients pre operatively.* All patients were operated by the senior author {ASG}. All patients were operated on a fracture table with boot traction using a direct lateral incision used for a conventional dynamic hip screw fixation.

### Insertion of the Richard’s screw

**Figure 1:**
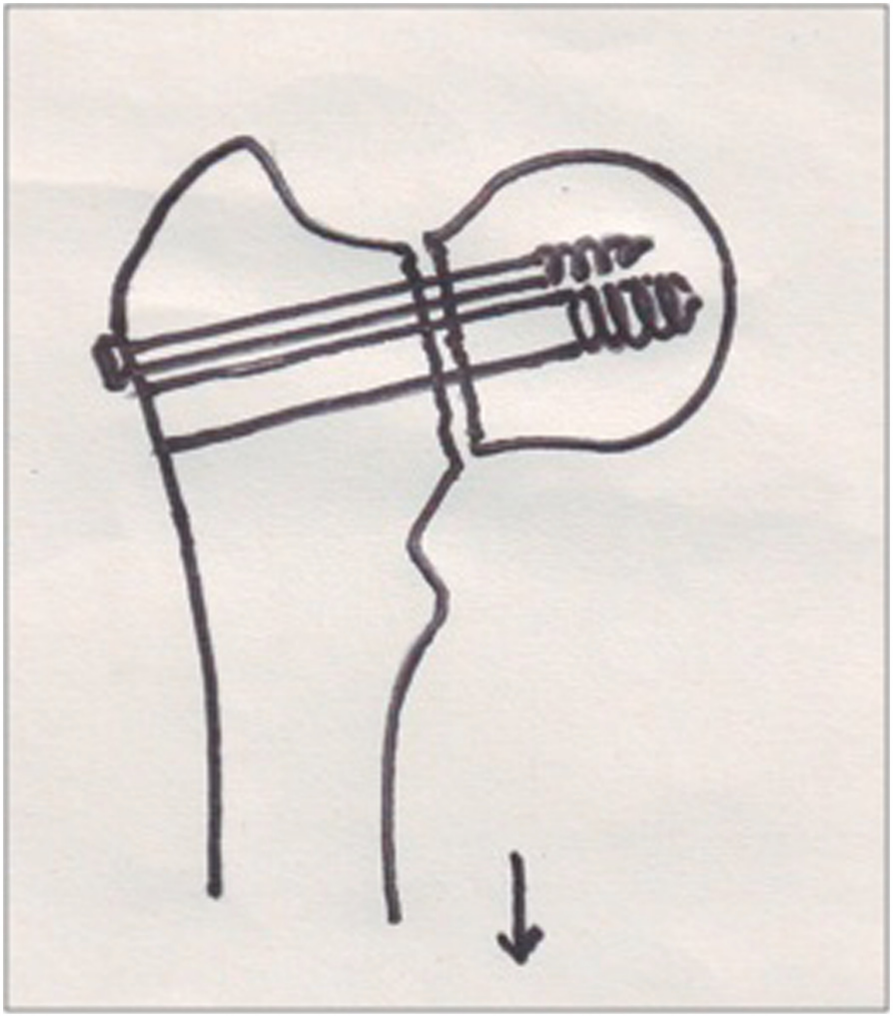
The derotation screw and the Richard’s screw are inserted along the axis of the femoral neck achieved on traction.

The 2.5 mm guide wire for the Richard’s screw was inserted such that it lies in the lower half of the femoral neck in AP view and in the center of the femoral neck in lateral view (Figure 1). *The guide wire was inserted along the axis of the femoral neck that has been achieved with traction.* After confirming the position of the guide wire in two planes, another 2.5 mm guide wire was passed superiorly to the first wire. A 6.5 mm partially threaded cancellous screw with a washer was inserted over the superior guide wire to prevent the head fragment from rotating during DHS insertion. The track for the DHS lag screw was created with a triple reamer and an appropriately sized lag screw was inserted.

### Technique of osteotomy and DHS fixation

**Figure 2:**
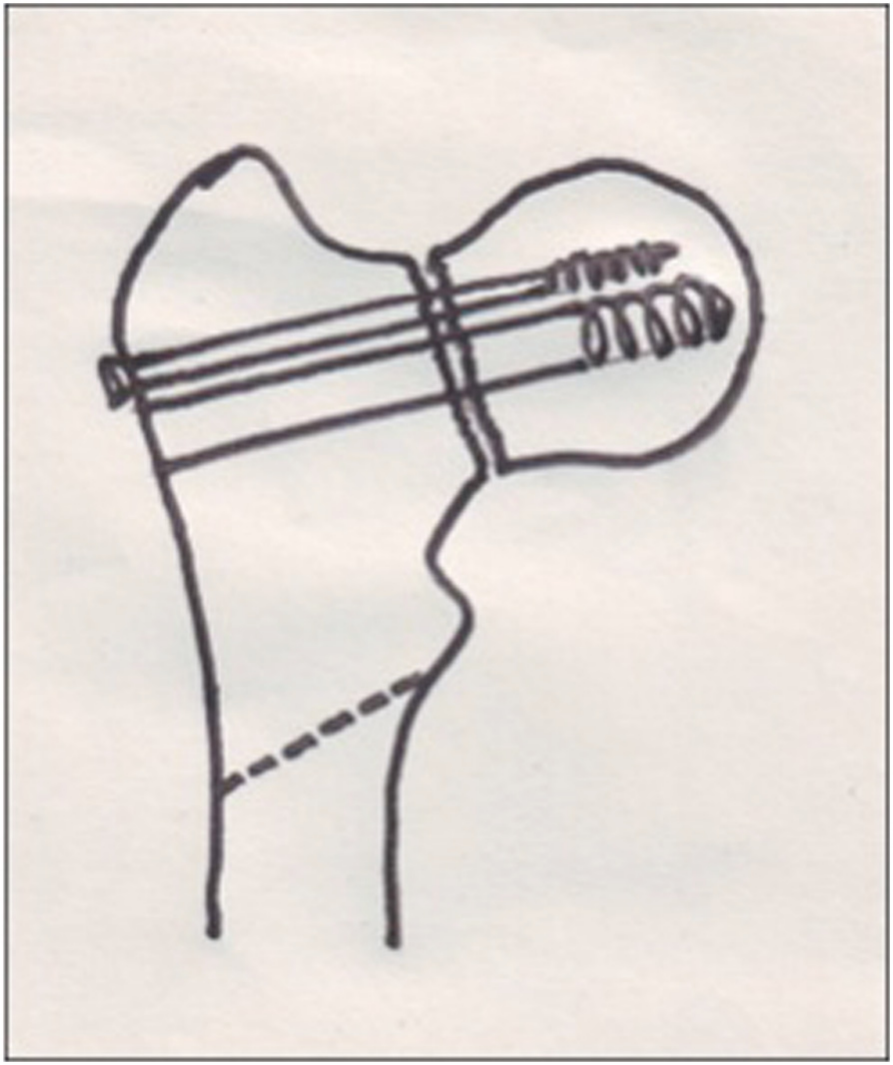
**The osteotomy is done in an oblique manner directed towards the base of the lesser trochanter.** The degree of obliquity depends on the neck shaft angle.

**Figure 3:**
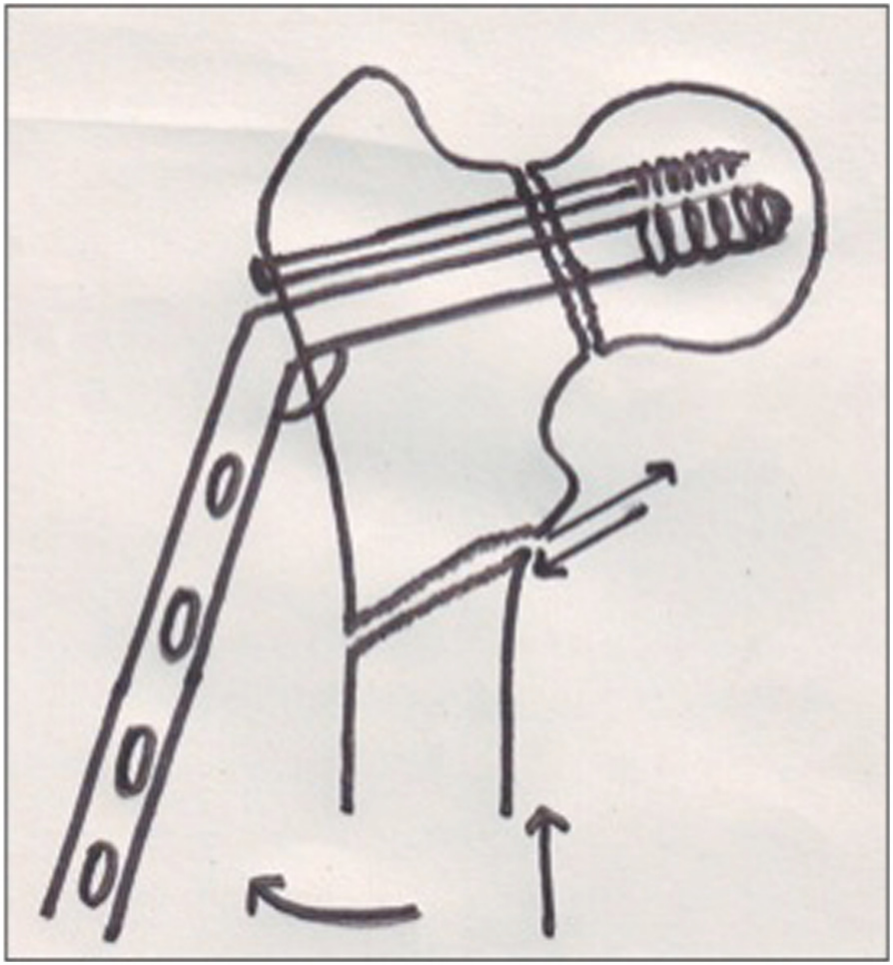
**The barrel plate doesn’t sit on the shaft because of the varus deformity.** Traction is released and the mobile distal fragment is abducted and secured to the plate using a plate holding clamp.

**Figure 4:**
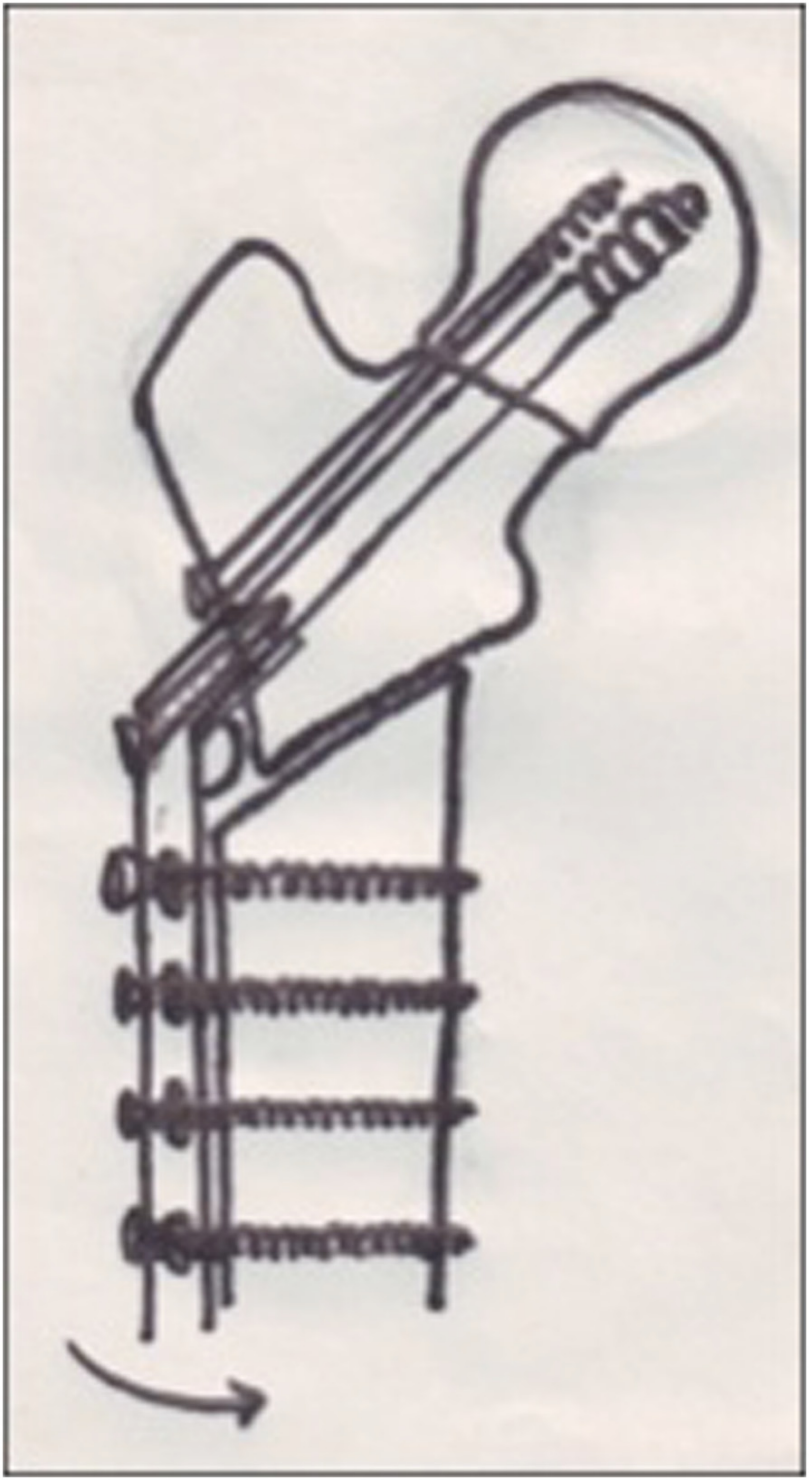
**The limb is brought to neutral.** The neck shaft angle is restored to the barrel plate angle and the fracture angle becomes more horizontal due to the combination of lateralisation of the distal fragment and valgus angulation of the proximal fragment.

*An oblique osteotomyw is done with an oscillating saw starting at the subtrochanteric region and directed towards the base of the lesser trochanter* (Figure 2 ).The pre-operative Pauwel’s angle minus 30 degrees, which was considered the desired Pauwel’s angle, gave the angle of the osteotomy. The 135 degrees barrel plate was then inserted over the lag screw. Since the lag screw has been inserted at much lesser angle, the 135 degree barrel plate will not sit on the lateral cortex (Figure 3). Traction was released and the limb was abducted in order to bring the shaft to the plate. Reduction of the shaft to the plate was done and maintained with a plate holding clamp. The limb was then brought to neutral with the barrel plate well secured to the bone to verify the neck shaft angle, Pauwel’s angle and the degree of lateralization of the distal fragment (Figure 4). The distal screws were inserted to secure the plate. The surgical technique was similar in both intra and extracapsular fractures, except that the derotational screw was not used for extracapsular fractures.

Thrombo embolic prophylaxis in the form of low molecular weight heparin was started 12 hours after surgery and was given till discharge. The average hospital stay was 6 days. All patients were mobilized immediately and were kept non weight bearing for 6 weeks and progressed to full weight bearing by 12 weeks. Patients were followed up regularly till fracture union. An ultimate follow up was arranged at one year to review hospital records and radiographs. Follow up radiographs were assessed by the authors for fracture union, corrections in Pauwel’s angle and neck shaft angle. Oxford hip score and limb length discrepancy were assessed at one year by two blinded trainees. The lowest clinically appreciable length discrepancy was set at 0.5 cm.

## Results

**Figure 5:**
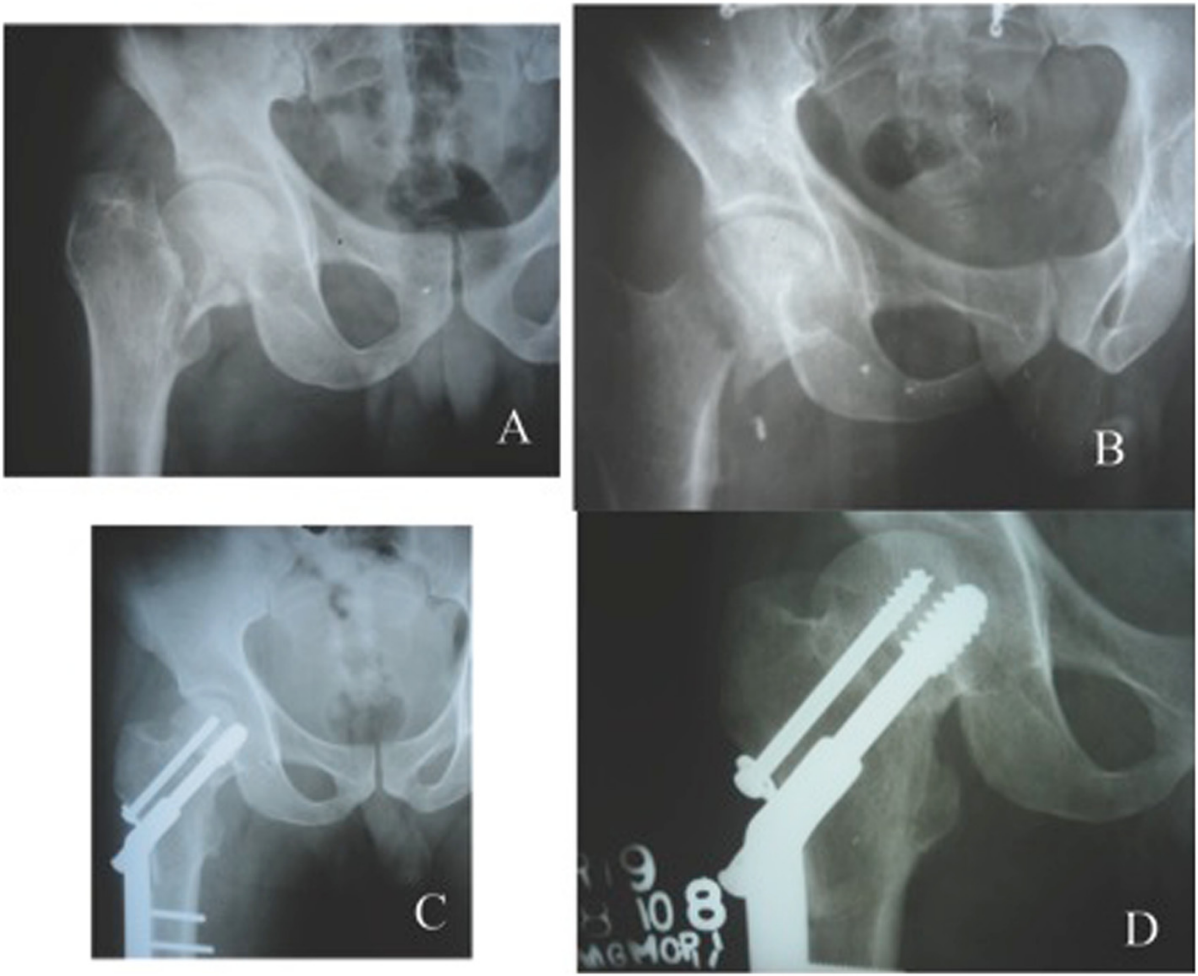
**Varus nonunion of an intracapsular neck fracture in a 37 year old male.** Post operative radiographs showing good union at the nonunion site and at the osteotomy site with restoration of the neck shaft angle.

**Figure 6:**
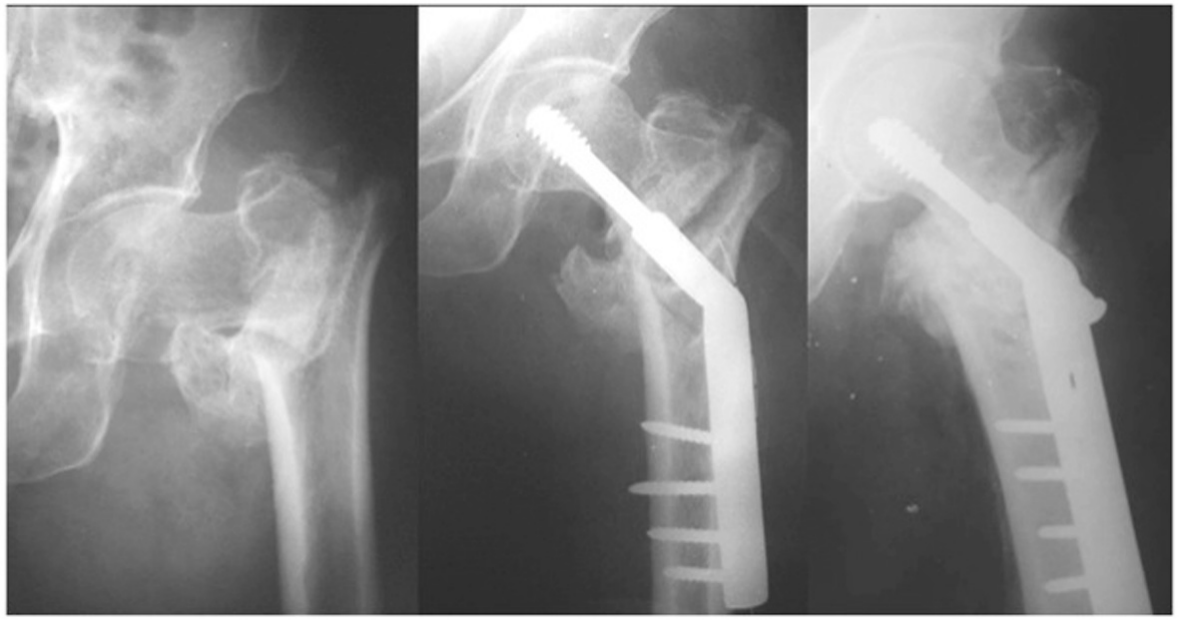
**Non union of a extracapsular fracture in exaggerated varus.** Good restoration of the neck shaft angle following oblique subtrochanteric osteotomy. Solid union is evident at the fracture and osteotomy sites.

The average surgical time was 70 minutes {55 – 90 minutes}. The average blood loss was 350 ml {250 – 600 ml}. All fractures united at a mean time of 10 weeks {8 – 13 weeks}. There was no x ray evidence of avascular necrosis at last follow up. The average pre operative Pauwel’s angle was 67 degrees {59–80 degrees} which was corrected to an average of 35 degrees {30–40 degrees}. The average correction in Pauwel’s angle was 32 degrees {22 – 46 degrees}. The mean neck shaft angle in pre operative traction films was 104 degrees {90 – 115 degrees} which was corrected to 136 degrees {130 – 140 degrees} achieving a mean correction of 32 degrees (Figures 5 and 6). The mean Oxford hip score improved from 20 {11 – 24} to 40 {38–42} at one year follow up. There were no screw cut outs, implant failure or loss of correction. There were no incidences of deep infection. 5 patients had clinically demonstrable limb shortening of 0.5 cm or more. None of the patients required a heel raise. 4 patients demonstrated mild trendelenberg lurch at last follow up. All patients resumed their normal activities at an average of 14 weeks and were able to walk unaided at last follow up. The results are summarized in Table 1.

**Table 1:** Patient data, radiological and functional outcome

**Patient**	**Age**	**Neck shaft angle score**		**Pauwel’s angle**		**Limb length discrepancy**		**Oxford hip**	
		**Pre op**	**Post op**	**Pre op**	**Post op**	**Pre op**	**Post op**	**Pre op**	**Post op**
1	31/M	105	138	62	34	1 cm	0	11	41
2	38/M	110	140	64	38	1.5 cm	0.7 cm	20	40
3	33/M	105	135	62	40	1.5 cm	0	22	40
4	44/M	105	137	80	34	1 cm	0	11	41
5	40/M	115	135	72	30	1 cm	0	19	39
6	28/M	108	140	66	36	1.5 cm	0.5 cm	21	40
7	37/M	116	135	59	36	1.5 cm	0.5 cm	20	38
8^*^	42/M	100	130	65	33	2cm	0	24	39
9^*^	55/M	95	135	60	35	2.5 cm	0.8 cm	23	42
10^*^	50/M	100	135	72	30	2 cm	0	23	40
11^*^	48/M	90	132	70	36	2 cm	0.7 cm	24	39
MEAN	41	104	136	67	35	1.60 cm		20	40

*: extracapsular neck fractures.

## Discussion

Different techniques of valgus angulation osteotomies have been described. All of them have two things in common; *extensive pre operative planning and removal of wedges from the proximal femur*. Our technique involves an oblique osteotomy just below the lesser trochanter and no wedges are taken. A greater degree of correction can be achieved with a more oblique osteotomy. A good area of contact at the osteotomy site can be maintained with an oblique osteotomy with minimal lateral displacement of the distal fragment and without significant opening up of the medial side as evident from Figures 5 and 6.

Subtrochanteric osteotomy was chosen to avoid compromising the lateral wall fragment with a proximal osteotomy especially in extracapsular fractures. A very low subtrochanteric osteotomy should be avoided as it will be through the cortical bone where union rates are less predictable and nonunion at the osteotomy site have been reported [3].

With an oblique osteotomy the distal fragment becomes free and can be laterally translated with ease along the osteotomy surface maintaining adequate contact with the proximal fragment. When the limb is brought to neutral, the neck shaft angle is restored to the barrel plate angle and the Pauwel angle at the fracture site is reduced due to the combination of valgus angulation of the proximal fragment and lateral displacement of the distal fragment*.*

Removing wedges may hinder limb length restoration and requires careful planning and templating to avoid the same. It also increases the surgical time and the blood loss.

Valgus osteotomy has a high success rate and good reproducibility among the augmentation techniques described for a femoral neck nonunion [4]. It is especially useful in younger individuals where a replacement surgery may not be the best option. Literature on nonunion of extracapsular fractures is sparse as these fractures commonly malunite rather than going for nonunion. While union in intracapsular fractures is complicated by biological and mechanical factors, the problem with extracapsular fractures is mainly mechanical. The applicability of valgus osteotomy in both intra and extracapsular fractures is based on similar principles.

Muller [5] has insisted on reducing the fracture angle to less than 25 degrees to achieve consistent results though other authors have reported otherwise. *We did not set any preliminary targets but aimed to bring the Pauwel’s angle to around 30 degrees with the obliquity of the osteotomy.* An osteotomy not only helps by improving the biomechanics but it also improves the hemodynamics at the nonunion site. A more horizontal fracture angle was achieved in all patients and they progressed to successful union. The limb shortening was brought to less than 1 cm in all patients. 4 patients had a trendelenberg lurch which may be due to the loss of abductor lever arm.

Valgus osteotomies using blade plates have been described by several authors [6-9]. Blade plates have excellent rotational control but are technically difficult to use. The dynamic hip screw is an excellent implant in this situation. It allows application of static compression during surgery with the coupling screw and also allows controlled dynamic collapse at the fracture site maintaining the neck shaft angle. The only drawback is the suboptimal rotational stability and rotational stress which may occur during reaming. Both these problems were addressed in our series to an extent by using an anti rotation screw along with the DHS in intracapsular fractures.

Recent articles on valgus osteotomies at the intertrochanteric level with DHS fixation has been described by Hartford et al., 2005 [10] and Schoenfeld et al., 2006 [11]. Both techniques in the own words of the authors required extensive pre operative sketching and templating to achieve the desired result. While Schoenfeld et al. removed a partial thickness wedge to minimise limb length discrepancy Hartford et al. used a full thickness laterally based wedge.

Schoenfeld et al. also noted that the partial thickness wedge osteotomy may decrease the surface area of contact and may increase the chances of implant failure. Though the post operative Pauwel’s angle was much lesser in their series, the final clinical, radiological and functional results were much similar to the current series. While Shoenfeld achieved a good limb length restoration with his technique, the mean post operative mean limb length discrepancy in the series by Hartford et al. was 1 cm probably because of the full thickness wedge technique they had used. Surgical time and blood loss were significantly lesser in our series compared to techniques described by these two authors.

To conclude, valgus osteotomy with DHS fixation is a useful technique for varus proximal femur nonunion in younger patients. Improving the biomechanics at the nonunion site coupled with a stable fixation yields good consistent results regarding union. The sliding osteotomy technique is simple, saves surgical time, minimises blood loss and helps in limb length restoration. No elaborate planning and wedge removal are required to achieve the desired results.

## Competing interests

The authors declare no competing interests.

## Authors’ contributions

ASG carried out the surgeries, follow up assessment and manuscript preparation. NCT carried out follow up assessments, manuscript editing and statistical analysis. All authors read and approved the final manuscript.
